# Supplementary material for: Fabrication of Multi-Material Pneumatic Actuators and Microactuators Using Stereolithography
Source: Micromachines (Basel). 2023 Jan 18;14(2):244. doi: 10.3390/mi14020244 (PMC9966499; doi:10.3390/mi14020244)
Supplement: Supplementary file 1 [file micromachines-14-00244-s001.zip › micromachines-2125401-supplementary.pdf]

Supplementary Materials

Fabrication of Multi-Material Pneumatic Actuators and Microactuators Using Stereolithography

Qingchuan Song, Yunong Chen, Peilong Hou, Pang Zhu, Dorothea Helmer, Frederik Kotz-Helmer * and Bastian E. Rapp

Table S1. Printer resolution and 3D part resolution of extrusion-based 3D printing methods (FDM, DIW, Polyjet) and stereolithography.

3D Printing Technology	Printer Resolution	3D Part Resolution	Source
FDM	0.4 mm	5 mm	[1]
DIW	0.1 mm	1.2 mm	[2]
Polyjet	0.016 mm	>0.75 mm	[3]
Stereolithography (this paper)	0.039 mm (xy)/ 0.010 mm (z)	~0.200 mm	-

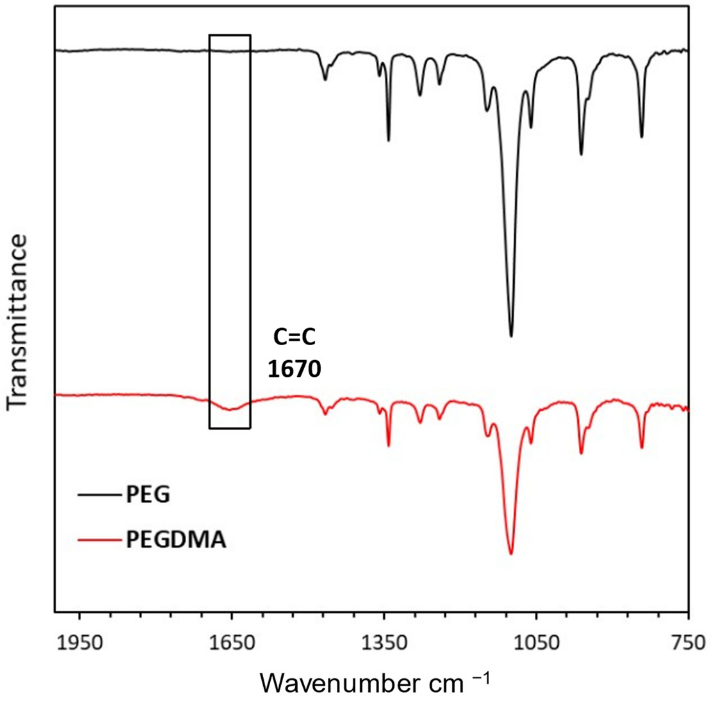

Figure S1. FTIR spectra of PEG10k (black) and PEGDMA10k (red). The peak at 1630–1730 cm^{-1} indicates the stretch of C=C bonds and C=O bonds, which were expected due to the introduction of the methacrylic group.

Video S1: Compression process of a multi-material lattice.

Video S2: Inflation process of a multi-material balloon.

Video S3: Bounce behavior of an inflated multi-material balloon.

Video S4: Assembly of multi-material actuators.

Video S5: Activation of fin-like microactuators.

References

1. Chacón, J.M.; Caminero, M.A.; García-Plaza, E.; Núñez, P.J. Additive Manufacturing of PLA Structures Using Fused Deposition Modelling: Effect of Process Parameters on Mechanical Properties and Their Optimal Selection. *Materials & Design* **2017**, *124*, 143–157.
2. Guo, S.-Z.; Qiu, K.; Meng, F.; Park, S.H.; McAlpine, M.C. 3D Printed Stretchable Tactile Sensors. *Adv. Mater.* **2017**, *29*, 1701218.
3. Ong, L.J.Y.; Islam, A.; DasGupta, R.; Iyer, N.G.; Leo, H.L.; Toh, Y.-C. A 3D Printed Microfluidic Perfusion Device for Multicellular Spheroid Cultures. *Biofabrication* **2017**, *9*, 045005.
